# Intelligent glucose management in hospitalized patients: Short-term glucose and adverse events prediction

**DOI:** 10.1371/journal.pone.0339360

**Published:** 2025-12-26

**Authors:** Jian Xue, Huaimin Cao, Lu Yang, Jiajia Liu, Ying Zhang, Chunlei Zhang, Ke Bai, Peng Jiang, Fanchang Hao, LiNa Xu

**Affiliations:** 1 School of Computer Science and Technology, Shandong Jianzhu University, Jinan, Shandong, China; 2 Department of Endocrinology, People’s Hospital of Gaotang County, Shandong, China; 3 Department of Medicine, People’s Hospital of Gaotang County, Shandong, China; 4 Digital Innovation Department, Beijing Intelligent Decision Medical Technology Co., LTD, Beijing, China; Kwara State University, NIGERIA

## Abstract

The management of blood glucose in hospitalized patients is confined to retrospective interventions, preventing healthcare professionals from predicting patients’ blood glucose levels and potential adverse events in advance. This study employs a deep learning model, specifically a Stacked Attention-Gated Recurrent Unit (SA-GRU) network, to forecast short-term blood glucose (BG) levels and predict adverse events in hospitalized patients, assisting clinicians in making clinical decisions. We collect continuous glucose monitoring(CGM) data from 196 hospitalized patients with type 2 diabetes, and by constructing and training this deep learning model, we predict blood glucose levels and adverse events.The model’s predictions are then compared with the actual CGM data, and different evaluation metrics are used to assess the predictions of blood glucose levels and adverse events. Additionally, experiments were conducted on another publicly available type 2 diabetes dataset. On our collected data, for the 30-minute prediction, the root mean square error (RMSE) and mean absolute relative difference (MARD) of blood glucose are 4.27 ± 0.31 mg/dL and 1.77% ± 0.08%, respectively, with an adverse event classification accuracy of 98.57% ± 0.11%. For the 60-minute prediction, the RMSE and MARD of blood glucose are 10.46 ± 0.55 mg/dL and 4.59% ± 0.22%, respectively, with an adverse event classification accuracy of 95.74% ± 0.33%. Similar positive results were obtained on another publicly available dataset. The proposed model demonstrates accurate predictions for blood glucose values and adverse events in the next 30 and 60 minutes.

## 1 Introduction

Diabetes, a prevalent metabolic disorder, is rapidly evolving into a significant global public health concern. According to the International Diabetes Federation (IDF), diabetes afflicted 10.5% of the worldwide adult population in 2021, and this figure is projected to rise. It is anticipated that by 2045, approximately 1 in 8 adults (equivalent to around 783 million individuals) will be affected by diabetes [1]. Diabetes exerts a profound influence on the health and overall quality of life of those impacted, and simultaneously imposes substantial strains on healthcare systems and socioeconomic structures.

Diabetes management plays a crucial role in the care of hospital patients. Many of these patients are admitted to the hospital due to complications related to diabetes or other health issues. During their hospital stay, healthcare professionals require continuous monitoring of their blood glucose levels to ensure optimal blood glucose control while receiving treatment and recovering. However, blood glucose in hospitalized patients can be influenced by factors such as dietary changes, adjustments in treatment regimens, and surgical procedures, which may lead to fluctuations in blood glucose and the occurrence of adverse events, including hypoglycemia and hyperglycemia. Hypoglycemia, hyperglycemia, and blood glucose fluctuations are strongly linked to adverse health outcomes, such as infections, cardiovascular complications, and increased mortality [2–6]. Hypoglycemia issues may result in higher treatment costs and pose significant health risks [7,8]. Hence, the implementation of intelligent blood glucose management for hospitalized patients is of paramount importance.

Traditional blood glucose monitoring methods have relied on multiple daily capillary point-of-care (POC) blood glucose tests to determine blood glucose [9]. However, recent years have witnessed remarkable advancements in diabetes monitoring technology, fundamentally transforming the management of blood glucose [10]. Continuous glucose monitoring (CGM) technology, which provides real-time data on blood glucose levels, is becoming increasingly popular. CGM not only offers real-time monitoring of blood glucose data but also provides immediate insights into blood glucose trends. This capability allows both patients and healthcare professionals to gain a deeper understanding of the patient’s blood glucose control status. Healthcare professionals widely acknowledge that CGM surpasses POC testing in its ability to prevent severe hyperglycemia and hypoglycemia [11,12]. CGM has already been integrated into blood glucose management decisions [13].

However, CGM technology has its limitations as it only provides current and past blood glucose data, lacking the ability to effectively predict future blood glucose levels. This deficiency may impede healthcare professionals from proactively intervening to avoid potential adverse events. With the rapid development of artificial intelligence (AI) technology, an increasing number of researchers are applying AI techniques in the medical field [14–17]. Predicting blood glucose levels and adverse events has become a feasible goal [18–20]. Leveraging the data from CGM and clinical decision support systems (CDSS), an AI-driven predictive model can be developed to intelligently foresee forthcoming blood glucose levels, thereby enabling early intervention in response to blood glucose levels and potential adverse events [21]. Such predictive models can be seamlessly integrated into CDSS, delivering real-time recommendations and guidance to healthcare professionals, assisting them in optimizing patients’ dietary plans, insulin dosages, and therapeutic choices. This, in turn, minimizes the risk of adverse events such as hyperglycemia and hypoglycemia, ultimately enhancing the quality and safety of patient care.

In this paper, we introduce a deep learning model designed for predicting blood glucose levels and the occurrence of adverse events in hospitalized patients with type 2 diabetes. The model leverages continuous glucose monitoring (CGM) data, which records blood glucose levels at regular intervals, typically every 15 minutes, over extended periods. These frequent, high-resolution measurements allow for precise short-term predictions of blood glucose fluctuations, as well as the identification of critical events such as hypoglycemia and hyperglycemia.

Unlike personalized glucose prediction methods that necessitate the division of individual patients’ glucose data into training and test sets, our approach divides the population into training and test sets. This eliminates the need for vast quantities of individual data, making the model more scalable and practical for real-world use in healthcare settings. Additionally, by focusing solely on blood glucose and time data, our model avoids the complexities and potential inaccuracies that could arise from incorporating other data types, such as dietary or medication information.

The primary contributions of this paper are as follows:

1. A deep learning model, Stacked Attention-Gated Recurrent Unit(SA-GRU), is presented by us, which intelligently forecasts short-term blood glucose levels and adverse events in hospitalized patients.
2. This model is trained using population-level data, obviating the requirement for extensive individual patient data. This feature enhances the scalability and practicality of the model.
3. The SA-GRU model exclusively relies on blood glucose and time data, mitigating the potential for error stemming from the inclusion of other data types during the collection process. Simultaneously, it simplifies the data collection process.

## 2 Method

### 2.1 Patient and public involvement

As the data were derived from patient records and publicly available datasets, there was no patient involvement.

### 2.2 Data sources and partitioning

This study is based on two datasets, namely the Gaotang dataset and the Shanghai dataset. The Gaotang dataset is a non-public dataset consisting of hospitalized patients with type 2 diabetes. Considering that validating the model on public datasets is a good way to demonstrate its effectiveness, we collected currently available public blood glucose datasets. Among them, the Shanghai dataset is closer to our research goal, but it consists of non-hospitalized patients with type 2 diabetes. Some other datasets either do not align with our research goals or cannot be used due to issues such as copyright and ethics.

The Gaotang dataset included 196 type 2 diabetes patients recruited from Gaotang County People’s Hospital in Shandong Province between 22/03/2023 and 27/05/2023. Detailed information is presented in Table 1. These patients were distributed across various departments including endocrinology, cardiology, neurology, orthopedics, and emergency medicine. The CGM was worn for durations ranging from 4 to 14 days, with blood glucose values recorded every 15 minutes, resulting in a total of 96 readings per day. The number of data recordings per patient ranged from 384 to 1340 times. The data was collected using the Abbott FreeStyle Navigator II subcutaneous CGM system, transferred to the CDSS system, and exported as structured data within the CDSS system. Data were accessed for research purposes in May 2023. The data collection process did not involve any information that could identify individual participants.

**Table 1:** Data distribution of Gaotang dataset.

Gender	Counts	Age	Hospital Stay (Days)	Data Points
Male	91	52.3±17.8	6.2±1.8	564±173
Female	105	58.8±15.2	6.4±2.0	584±191

In terms of inclusion and exclusion criteria, only hospitalized patients diagnosed with type 2 diabetes and having available continuous glucose monitoring (CGM) data were included. Patients wearing the Abbott FreeStyle Navigator II CGM system for a period of 4–14 days during the data collection period were considered eligible. Exclusion criteria involved patients with significant data loss (e.g., interruptions greater than 1 day) or abnormal glucose readings (e.g., values of 0 or extreme outliers).

Researchers who require access to this dataset can contact us via our dedicated email, gaotangglucosedataset@gmail.com and we will send the data access request procedure via email.

The Shanghai dataset collected clinical data from diabetes patients who sought medical treatment at two hospitals in Shanghai from 2019 to 2021 [22]. For this study, we specifically selected data from 109 cases of type 2 diabetes patients, as outlined in Table 2. Data collection utilized the Abbott FreeStyle Navigator II subcutaneous CGM system, with the number of data recordings per patient ranging from 247 to 1339 times.

**Table 2:** Data distribution of Shanghai dataset.

Gender	Counts	Age	Hospital Stay (Days)	Data Points
Male	59	59.3±14.9	10.5±3.7	1006±351
Female	50	61.5±13.5	11.0±3.3	1059±318

### 2.3 Data preprocessing

#### 2.3.1 Unit transformation and normalization.

To enhance the model’s focus on the relationship between different times of the day and blood glucose levels, we incorporated time as a feature within the model alongside blood glucose levels. We conducted unit transformation and normalization on the two features, blood glucose values and time, with the aim of facilitating comparisons with other studies and expediting the convergence of the model’s loss function.

Specifically, we converted the unit of blood glucose values from mmol/L (millimoles per liter) to mg/dL (milligrams per deciliter). This unit conversion ensures that our results can be globally compared with other relevant studies.

Convert the time format recorded by CGM from hh:mm to a decimal format. If the hour part remains unchanged, the minute part is converted to a decimal part by dividing the minutes by 60. For example, 11:30 is converted to 11.5.

Data normalization is a common data preprocessing technique in data analysis and mining, which has been widely applied in various fields including healthcare [23,24]. Equation 1 represents a common method of data normalization. Its main purpose is to transform data with different scales, units, or ranges into a uniform standard, thereby eliminating dimensional and size differences among the data, improving the performance of machine learning models, accelerating the convergence speed of models, and avoiding the influence of outliers on the model [25].


$$X^{\prime }=\frac{X-X_{min}}{X_{max}{-X}_{min}}$$ (1)

As depicted in Equation 1, we employed min-max normalization to standardize all blood glucose data to a range between 0 and 1. In this Equation, X represents the CGM blood glucose value, $X_{min}$ corresponds to the smallest blood glucose value among all blood glucose data, and $X_{max}$ represents the largest blood glucose value among all the data. To encompass the blood glucose ranges of both datasets, $X_{min}$ and $X_{max}$ were respectively set to the maximum values in the two datasets, 500.4 mg/dL and 39.6 mg/dL. According to the definition by the European Association for the Study of Diabetes (EASD), the first-level hypoglycemia range is defined as Glucose <70 mg/dL (<3.9 mmol/L) and ≥54 mg/dL (≥3.0 mmol/L), while the second-level hypoglycemia is defined as Glucose <54 mg/dL (<3.0 mmol/L). Hyperglycemia is defined as Glucose ≥180 mg/dL (≥10.0 mmol/L) [26]. The range we selected is well below and above the lowest and highest standards of the normal blood glucose range. If values exceed this range, immediate action by a physician is necessary.

#### 2.3.2 Time step construction.

**Fig 1:**
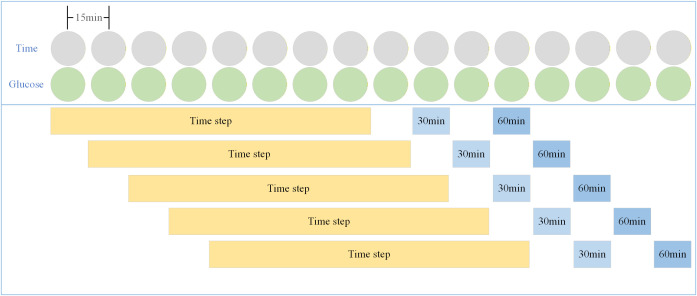
Time step construction.

To determine the optimal time interval for continuous data, we found that a 120-minute interval yielded higher accuracy. CGM blood glucose data collection is conducted at 15-minute intervals, and we have set the length of each step to 8 to ensure that each time step represents 120 minutes. These time steps encompass both types of data, namely blood glucose values and the corresponding timestamps. Concretely, the generation of these time steps involves sequential sliding starting from the initial data point. We designate every eight data points as a distinct time step. The subsequent model updates are based on these continuous two-hour measurements, rather than using model-predicted data to continuously update the model. This is because predicted values may introduce biases, and incorporating them as new data inputs could potentially amplify the model’s predictive biases. The process is visually depicted in Fig 1, where the yellow boxes represent the time steps, and the blue boxes signify the predicted values.

### 2.4 Structure of SA-GRU model

**Fig 2:**
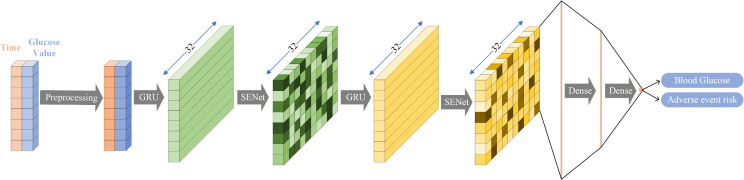
Architecture of SA-GRU.

The architecture of the SA-GRU model is depicted in Fig 2, with detailed information presented in Table 3, where Fully Connected (FC) denotes the fully connected network. Before feeding the data into the deep learning network, it undergoes preprocessing. Subsequently, the model predicts both blood glucose and adverse events, with adverse event identification based on single-point thresholds for hypo- and hyperglycemia, using the predicted blood glucose levels at each time point and predefined threshold values to determine the occurrence of such events.

**Table 3:** Detailed parameters of SA-GRU.

Layer	Layer Description	Output Batch × Steps × Channels	Hidden layer
1	GRU	512x8x32	32
2	SENET	512x8x32	–
3	GRU	512x8x32	32
4	SENET	512x8x32	–
		Batch × Channels	
5	FC1	512x128	–
6	FC2	512x32	–
7	FC3	512x1	–

The 2D array, consisting of time points and corresponding blood glucose values, is fed into the GRU model for feature extraction. The model then generates an output with the shape (batch size, time steps, hidden size), where time steps represents the data’s time window, and hidden size refers to the size of the GRU’s hidden layer. This output contains multiple feature channels, each capturing distinct patterns from blood glucose variations, such as basal glucose levels and postprandial fluctuations.

SENet applies an attention mechanism to the feature channels, allowing the model to dynamically adjust the importance of each feature at different time steps. Specifically, we first swap the dimensions of the data, transforming the shape from (batch size, time steps, hidden size) to (batch size, hidden size, time steps). Then, SENet calculates attention weights along the feature channel dimension. This process enables the model to reweight each feature channel based on its relevance, highlighting critical features for predicting blood glucose changes. Finally, the attention-weighted features are restored to the original time step dimension, ensuring that the model makes accurate predictions by taking into account both temporal and feature importance.

Based on our experimental experience and comparisons of different models, we chose a two-layer stacked GRU as the model architecture. Through experiments, we found that adding more layers to the GRU could slightly increase the model’s learning capacity but did not show a significant improvement in model performance. On the contrary, increasing the number of layers could lead to excessive model parameters, increasing the risk of overfitting, as well as increasing training time and computational costs. Therefore, we believe that a two-layer GRU is sufficient to capture the features of the data well while maintaining a simple model structure.

#### 2.4.1 Gated recurrent unit.

Recurrent neural networks (RNN) [27] find extensive application in handling time series data, such as blood glucose prediction, where they play a crucial role in appropriately extracting temporal information. Among the commonly used RNN variants, long short-term memory (LSTM) [28] and gated recurrent unit (GRU) [29] stand out. Both of these variants are designed to address the gradient problem encountered in traditional RNN, but they differ in structure. The GRU distinguishes itself by reducing the number of parameters and computational complexity, resulting in a more compact and simplified architecture. This characteristic endows GRUs with a unique advantage—being both accurate and computationally efficient, especially when working with large training datasets. This advantage is crucial in blood glucose prediction tasks that require high accuracy and high real-time performance.

**Fig 3:**
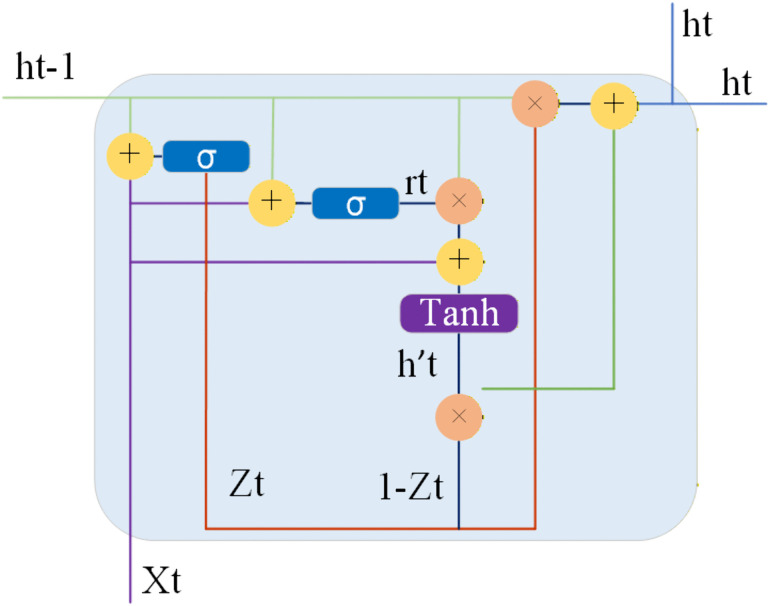
Architecture of GRU.

The GRU comprises the update gate and the reset gate, both of which enable the GRU to effectively capture dependencies across different moments in time series data. Through the reset gate, the model can control the degree to which previous state information is retained, while the update gate determines the extent of influence of the previous state on the current one. These mechanisms allow the GRU to flexibly adjust memory and forgetting when processing time series data. Its structure is illustrated in Fig 3.

$x_{t}$ represents the input information at the current time step, while $h_{t-\text{1}}$ represents the output information from the previous time step. $r_{t}$ and $z_{t}$respectively denote the reset gate vector and the update gate vector. When $x_{t}$ and $h_{t-\text{1}}$ are inputted into the network, they are multiplied by their respective weights $W^{\left(r\right)}$and $U^{\left(r\right)}$, then added together, and finally compressed through a sigmoid function to ensure the output values are between 0 and 1. This process is illustrated in Equation 2.


$$r_{t}=\sigma (W^{\left(r\right)}x_{t}+U^{\left(r\right)}h_{t-\text{1}})$$ (2)

Subsequently, the gated recurrent unit computes a candidate hidden state to prepare for the calculation of the subsequent hidden state. The output of the reset gate $r_{t}$ at the current time step is element-wise multiplied by the previous time step’s hidden state $h_{t-\text{1}}$. Then, it is combined with the weighted input $x_{t}$ of the current time step, $W^{\left(h\right)}$ being the weight of $x_{t}$. Finally, the candidate hidden state is compressed between −1 and 1 through the activation function tanh. This generates the candidate hidden state $h_{t}^{\prime }$ for the current time step, as illustrated in Equation 3.


$$h_{t}^{\prime }=tanh({W^{\left(h\right)}x}_{t}+{r_{t}⊙h}_{t-\text{1}})$$ (3)

The update gate determines the extent of influence of the previous state on the current one. A higher value of the update gate indicates a more significant influence of the previous state on the current state. Specifically, the update gate helps the model determine how much information from past time steps needs to be passed to future time steps. This process, as illustrated in Equation 4, is similar to the reset gate. In this Equation, when $x_{t}$ and $h_{t-\text{1}}$ are inputted into the network, they are multiplied by their respective weights $W^{\left(z\right)}$and $U^{\left(z\right)}$, added together, and finally compressed through a sigmoid function to obtain output values between 0 and 1.


$$z_{t}=\sigma (W^{\left(z\right)}x_{t}+U^{\left(z\right)}h_{t-\text{1}})$$ (4)

Finally, by combining the update gate $z_{t}$ at the current time step, the previous time step’s hidden state $h_{t-\text{1}}$ is combined with the current time step’s candidate hidden state $h_{t}^{\prime }$. This process is illustrated in Equation 5.


$$h_{t}=z_{t}⊙h_{t-\text{1}}+(\text{1}-z_{t}){\;⊙\;h}_{t}^{\prime }$$ (5)

When the data passes through all hidden layers in the GRU, the output is denoted as K.

#### 2.4.2 Squeeze-and-excitation network.

**Fig 4:**
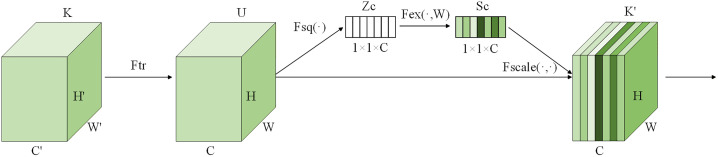
Architecture of SENet.

The Squeeze-and-Excitation Network (SENet) [30] is an attention mechanism used in CNNs, aimed at enhancing the network’s representation capability on feature channels. It has wide applications in image classification and feature extraction tasks. It enhances the representation of useful information and suppresses irrelevant information by learning the importance weights of each channel and then reweighting the feature maps accordingly. SENet consists of two key stages: the squeeze stage and the excitation stage. Its structure is illustrated in Fig 4.

$F_{tr}$ represents the transformation function, typically implemented using convolutional operations. Its role is to transform the input data K into the output data U, where K is the input feature map and U is the output feature map. Through convolutional operations, $F_{tr}$ applies the learned filter kernels to the input data K to generate the output data U.

In the compression stage, the input feature maps undergo global average pooling. Specifically, for feature maps with shape (C, H, W), global average pooling yields a tensor of size (C, 1, 1), where C is the number of channels, and H and W are the height and width, respectively. This process is illustrated in Equation 6.


$$Z_{c}=F_{sq}\left(K_{c}\right)=\frac{\text{1}}{H*W}\sum {\mathrm{\backslash nolimits}}_{i=\text{1}}^{H}{sum}_{j=\text{1}}^{W}U_{c}(i,j)$$ (6)

In the excitation stage, the importance weights of the channels are learned through a two-layer fully connected network. This process is depicted in Equation 7, where δ denotes the ReLU function, $W_{\text{1}}$ and $W_{\text{2}}$ are weight matrices, and σ represents the sigmoid function.


$$S_{c}=F_{ex}\left(Z,W_{i}\right)=\sigma \left(g\left(Z,W_{i}\right)\right)=\sigma \left(W_{\text{2}}\delta \left(W_{\text{1}}Z\right)\right)$$ (7)

The result of the excitation stage is finally multiplied element-wise with the original feature maps, resulting in weighted feature maps for each channel. This process, shown in Equation 8, enables the network to better learn important information from each channel, thereby enhancing the network’s performance in feature extraction.


$$K^{\prime }=F_{scale}\left(U_{c},S_{c}\right)=S_{c}U_{c}$$ (8)

The input data passes through the first GRU module to produce K, which then goes through SENet to output $K^{\prime }$. Afterward, it undergoes another GRU module to output $K^{\prime \prime }$. Similarly, after passing through SENet again, it outputs . At this point, this result is fed into the fully connected network.

### 2.5 Model evaluation indicators

In this paper, we employ a range of evaluation metrics to assess the model’s performance. In the context of blood glucose prediction, we utilize the following metrics: RMSE, mean absolute error(MAE), MARD, and the Clark Error Grid Analysis. Among these, RMSE, MAE, and MARD evaluate the model’s error in predicting blood glucose compared to CGM-measured blood glucose, while the Clark Error Grid Analysis assesses the severity of error events. For the evaluation of adverse events prediction, we employ Accuracy, Precision and Recall. To classify blood glucose levels, we established the following criteria: blood glucose values less than 70 mg/dL are categorized as hypoglycemic, values greater than 180 mg/dL fall within the hyperglycemic range, and values between 70 mg/dL and 180 mg/dL are considered normal. Adverse events are identified if a patient’s blood glucose level falls into either the hyperglycemic or hypoglycemic range.

RMSE: The root mean square error between the predicted and CGM blood glucose values is described in equation 9, where $Y^{\prime }$ represents the predicted blood glucose value, Y represents the true blood glucose value, and n represents the total number of samples in the validation set.


$$RMSE=\;\sqrt{\frac{\sum_{i=\text{1}}^{n}{\left({Y_{i}}^{\prime }-Y_{i}\right)}^{\text{2}}}{n}}$$ (9)

MAE: The mean absolute error is a metric used to quantify the average magnitude of errors between the predicted and actual values. It is defined by equation 10, where $Y^{\prime }$ represents the predicted blood glucose value, Y represents the true blood glucose value, and n is the total number of samples in the validation set.


$$MAE=\;\frac{\text{1}}{n}\sum {\mathrm{\backslash nolimits}}_{i=\text{1}}^{n}\left|{Y_{i}}^{\prime }-Y_{i}\right|$$ (10)

MARD: The mean absolute relative difference between the predicted and CGM blood glucose values is described in equation 11. A smaller percentage in the MARD value indicates a closer match between the predicted value and the CGM value, while a larger MARD percentage signifies a greater difference between the predicted value and the CGM value [31].


$$MARD=\;\frac{\text{1}}{n}\sum {\mathrm{\backslash nolimits}}_{i=\text{1}}^{n}\left|\frac{{Y_{i}}^{\prime }-Y_{i}}{Y_{i}}\right|*\text{1}\text{0}\text{0}\%$$ (11)

The Clark Error Grid Analysis is a method used to evaluate the performance of blood glucose prediction models and is commonly used to evaluate the accuracy of CGM devices or blood glucose prediction algorithms [32].

Accuracy: Accuracy is the classification accuracy of the model, i.e., how often the model correctly predicts adverse events. A high accuracy rate indicates that the model is more likely to distinguish adverse events with a higher probability, thus improving the accuracy of adverse events predictions.

Precision: Precision refers to the model’s ability to accurately identify and classify instances as positive. It signifies the proportion of correctly predicted positive instances among all instances predicted as positive by the model. A high precision rate indicates that the model less frequently makes errors in predicting negative instances as positive.

Recall: Recall is the rate at which the model can correctly identify adverse events. A high recall rate indicates that the model rarely misses any adverse events, reducing the risk of false negatives.

### 2.6 Model parameters and training

The experiment utilized the Pytorch deep learning framework with Python version 3.9, CUDA version 10.2, an NVIDIA GeForce RTX 2080 Ti GPU, and 11 GB of graphics memory. The model parameters were set as follows: batch size = 512, learning rate = 0.001, epoch = 500, and the Adam optimizer was employed.

The SA-GRU model consists of a total of 31,557 parameters. These parameters represent various weights and biases within the model, constituting the structure and functionality of the model. Specifically, these parameters cover different levels and nodes of the model, including input, hidden, and output layers, as well as various connection weights and bias terms.

In each experiment, we randomly divided the dataset into training, validation, and test sets in a 6:2:2 ratio. Additionally, the data was inspected before being inputted into the model to ensure that there was no overlap between the datasets. In the Gaotang dataset, each randomly split training set contained data from 118 patients, both the validation and test sets each contained data from 39 patients. The data for each patient ranged from 384 to 1340 readings. In the Shanghai dataset, each randomly split training set contained data from 65 patients, both the validation and test sets each contained data from 22 patients. The data for each patient ranged from 247 to 1339 readings. We chose this split ratio to ensure an adequate amount of training data while providing sufficient validation and test data to evaluate the model’s generalization ability.

To reduce randomness and enhance the robustness of model performance evaluation, we conducted ten repetitions of validation. In each validation iteration, we redivided the dataset and executed the process of model training, validation, and testing to ensure full utilization of the data and obtain reliable evaluation results. This validation approach helps mitigate the effects of data randomness, thereby increasing the credibility and robustness of model evaluation.

## 3 Result

### 3.1 Model performance

Our work involves constructing population-based models, offering the advantage of not requiring extensive individual data. In contrast, other models typically demand substantial individual data for high precision, often necessitating data spanning at least several days to ensure accuracy. Our approach can accurately predict blood glucose levels based on just 2 hours of patient data. For predictive tasks involving large populations, our proposed method exhibits greater scalability and practicality. Meanwhile, in this study, our model demonstrates low computational burden during inference. Testing on an NVIDIA GeForce RTX 2080 Ti GPU, the model achieved an inference time of just 4.52 ms per sample, ensuring real-time performance when processing blood glucose data. This rapid inference capability enables timely predictions, making the model suitable for real-time clinical use and enhancing its practicality in fast-paced healthcare settings.

Although direct comparisons with other models are not feasible, to evaluate our model’s performance, we selected several classic benchmark models such as support vector regression(SVR) [33], eXtreme Gradient Boosting(XGBOOST) [34], ANN, CNN, LSTM, and GRU. Additionally, to further validate the effectiveness of our work, we conducted ablation experiments comparing the original GRU, stacked GRU (excluding SENet), and our proposed SA-GRU. Tables 4 and 5 present the blood glucose prediction results and adverse event prediction results of different models on the Gaotang dataset across various time intervals. Similarly, Tables 6 and 7 depict the results for the Shanghai dataset.

**Table 4:** Performance of different models to predict blood glucose values on the Gaotang dataset.

	30min			60min		
	RMSE	MAE	MARD	RMSE	MAE	MARD
SVR	13.30 ± 1.40	11.63 ± 1.38	9.04 ± 1.31	18.18 ± 0.38	15.38 ± 0.44	11.79 ± 0.52
XGBOOST	12.14 ± 0.63	8.42 ± 0.32	5.72 ± 0.27	20.04 ± 0.69	14.24 ± 0.44	9.48 ± 0.46
ANN	5.33 ± 0.25	3.41 ± 0.12	2.28 ± 0.07	12.91 ± 0.35	8.66 ± 0.19	5.77 ± 0.21
CNN	4.51 ± 0.45	2.85 ± 0.33	1.86 ± 0.24	10.66 ± 0.59	7.19 ± 0.39	4.78 ± 0.29
LSTM	4.57 ± 0.45	2.83 ± 0.22	1.85 ± 0.15	11.12 ± 0.52	7.40 ± 0.27	4.84 ± 0.20
GRU	4.52 ± 0.24	2.78 ± 0.06	1.82 ± 0.06	10.88 ± 0.55	7.17 ± 0.31	4.80 ± 0.14
SA-GRU	**4.27 ± 0.31**	**2.64 ± 0.15**	**1.77 ± 0.08**	**10.46 ± 0.55**	**6.91 ± 0.35**	**4.59 ± 0.22**

* The units of RMSE and MAE are mg/dL and the units of MARD are %.

**Table 5:** Performance of different models to predict adverse events on the Gaotang dataset.

	30min			60min		
	Accuracy	Precision	Recall	Accuracy	Precision	Recall
SVR	93.76 ± 0.86	93.89 ± 0.66	72.08 ± 4.20	91.70 ± 0.60	92.17 ± 1.48	65.24 ± 0.57
XGBOOST	94.77 ± 0.22	90.28 ± 0.81	82.30 ± 1.68	91.29 ± 0.80	80.78 ± 11.1	69.86 ± 6.11
ANN	97.88 ± 0.12	95.70 ± 0.76	93.55 ± 1.29	94.42 ± 0.29	88.77 ± 1.31	84.36 ± 2.30
CNN	98.28 ± 0.25	96.05 ± 0.76	94.76 ± 1.09	95.47 ± 0.27	90.55 ± 2.41	86.96 ± 1.80
LSTM	98.21 ± 0.20	95.80 ± 0.99	95.20 ± 0.61	95.41 ± 0.32	89.25 ± 2.07	**89.03 ± 1.56**
GRU	98.26 ± 0.19	95.36 ± 0.77	**95.62 ± 0.70**	95.34 ± 0.28	90.52 ± 1.38	88.93 ± 2.00
SA-GRU	**98.57 ± 0.11**	**96.42 ± 0.53**	94.97 ± 0.96	**95.74 ± 0.33**	**90.60 ± 2.09**	88.62 ± 1.74

* All units in the table are %.

**Table 6:** Performance of different models to predict blood glucose values on the Shanghai dataset.

	30min			60min		
	RMSE	MAE	MARD	RMSE	MAE	MARD
SVR	18.08 ± 0.55	12.30 ± 0.31	8.95 ± 0.28	28.17 ± 0.63	22.40 ± 0.56	18.87 ± 1.02
XGBOOST	17.86 ± 0.44	14.91 ± 0.45	12.70 ± 0.99	27.23 ± 1.15	19.15 ± 0.87	14.29 ± 1.03
ANN	15.87 ± 4.31	11.37 ± 4.07	8.50 ± 3.19	26.56 ± 1.62	18.39 ± 1.21	13.28 ± 0.98
CNN	13.63 ± 0.95	9.62 ± 0.68	7.43 ± 0.61	24.86 ± 1.14	17.36 ± 0.83	12.38 ± 0.78
LSTM	13.88 ± 1.47	9.82 ± 1.29	7.36 ± 1.21	24.11 ± 1.16	16.80 ± 0.73	12.43 ± 0.64
GRU	13.65 ± 0.42	9.56 ± 0.23	7.12 ± 0.26	23.81 ± 1.21	16.47 ± 0.89	11.97 ± 0.39
SA-GRU	**13.56 ± 0.81**	**9.55 ± 0.52**	**6.88 ± 0.33**	**23.70 ± 1.48**	**16.40 ± 0.88**	**11.91 ± 0.64**

* The units of RMSE and MAE are mg/dL and the units of MARD are %.

**Table 7:** Performance of different models to predict adverse events on the Shanghai dataset.

	30min			60min		
	Accuracy	Precision	Recall	Accuracy	Precision	Recall
SVR	92.41 ± 0.64	78.74 ± 6.48	65.09 ± 2.27	87.97 ± 0.90	70.02 ± 6.6	56.08 ± 1.37
XGBOOST	91.94 ± 0.91	**88.41 ± 4.39**	68.61 ± 4.25	88.48 ± 1.68	63.6 ± 11.31	54.6 ± 3.34
ANN	92.52 ± 3.27	82.73 ± 5.03	80.91 ± 7.15	88.45 ± 1.23	72.81 ± 4.73	63.47 ± 5.08
CNN	93.81 ± 0.68	85.51 ± 3.89	81.11 ± 2.73	88.94 ± 0.93	75.42 ± 7.39	64.52 ± 4.09
LSTM	93.95 ± 0.83	85.10 ± 4.14	82.55 ± 3.38	89.27 ± 0.96	**77.01 ± 3.72**	68.67 ± 4.09
GRU	93.39 ± 0.49	86.74 ± 4.13	81.46 ± 2.49	89.95 ± 0.77	75.72 ± 4.13	68.57 ± 4.99
SA-GRU	**94.01 ± 0.53**	84.66 ± 4.31	**82.66 ± 3.22**	**90.09 ± 0.77**	75.32 ± 5.87	**69.36 ± 4.14**

* All units in the table are %.

From the experimental results on two different datasets, we observe overall consistency in the performance of these models across both datasets, with machine learning models generally lagging behind deep learning models. Notably, among the deep learning models, non-temporal models such as ANN and CNN exhibit inferior performance compared to temporal data models. In a comprehensive comparison, the SA-GRU model surpasses other models. The results on the Shanghai dataset, relative to the Gaotang dataset, show higher overall errors and lower accuracy. This is mainly because the Shanghai dataset consists of non-hospitalized patients with type 2 diabetes. As mentioned earlier, there are currently no publicly available datasets for hospitalized patients with type 2 diabetes. To validate the effectiveness and performance of the model, we conducted experiments using a dataset of non-hospitalized patients with type 2 diabetes, which shares similar features.

To comprehend the efficacy of each network component, we conducted experiments on models with different modules and evaluated their performance. The results, as depicted in Table 8, demonstrate that the SA-GRU model attains the best performance, affirming the effectiveness of SENet and the stacked model.

**Table 8:** Model ablation experiments on the Gaotang dataset.

	30min		60min	
	RMSE(mg/dL)	Accuracy(%)	RMSE(mg/dL)	Accuracy(%)
GRU	4.52 ± 0.24	98.26 ± 0.19	10.88 ± 0.55	95.34 ± 0.28
Stacked GRU	4.49 ± 0.24	98.46 ± 0.12	10.59 ± 0.52	95.60 ± 0.38
SA-GRU	4.27 ± 0.31^**^	98.57 ± 0.11^*^	10.46 ± 0.55^**^	95.74 ± 0.33^*^

To statistically confirm the observed improvements, we performed paired t-tests on the 10-fold cross-validation results for both RMSE and accuracy at 30-minute and 60-minute intervals. The t-tests were used to assess whether the differences in performance between the models were statistically significant. The results indicate that SA-GRU significantly outperforms both the GRU models, as reflected in the RMSE and accuracy metrics at both intervals. Specifically, * indicates p < 0.05 and ** indicates p < 0.01. These statistical findings further reinforce that SA-GRU provides substantial performance improvements compared to the baseline models.

### 3.2 Visualization of experimental results

**Fig 5:**
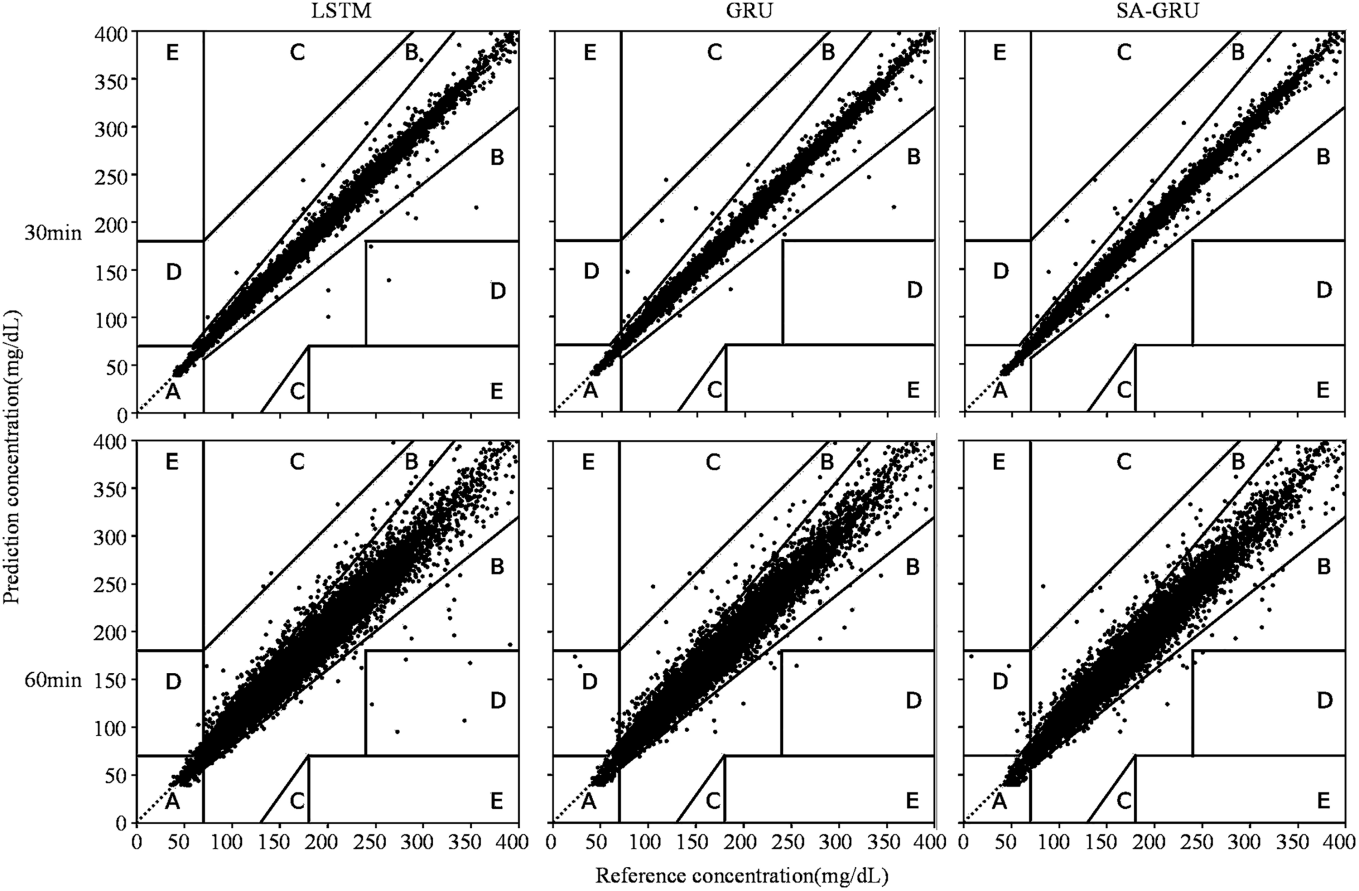
Clark error grid (Gaotang dataset models).

In Fig 5, the Clark grid error analysis shows the performance of different models on the Gaotang dataset. The horizontal axis represents the blood glucose values from CGM, while the vertical axis represents the blood glucose values predicted by the model. Each data point on the graph corresponds to a specific time, depicting CGM blood glucose value against the predicted value. The grid employed in the figure classifies blood glucose values into five distinct regions, each with its own significance:

Region A signifies that the variance between predicted and actual values has no clinical impact, representing the ideal scenario.

Region B indicates a minor disparity between predicted and actual values, which may have some influence on clinical decision-making but typically does not pose a substantial risk to the patient.

Region C highlights a significant deviation between the predicted and actual values, potentially jeopardizing the patient’s well-being and leading to erroneous treatment decisions.

Region D denotes that the predicted value exceeds the actual blood glucose level, potentially resulting in unwarranted treatment for the patient.

Region E signifies that the predicted value falls short of the actual blood glucose level, potentially leading to inappropriate treatment for the patient.

Within this context, the SA-GRU model exhibits outstanding performance in predicting blood glucose levels, both for the upcoming 30 minutes and the subsequent 60 minutes. At the 30-minute mark, all predicted data points are contained within the A and B regions, with 9773 data points in Zone A, 15 in Zone B, and 0 in Zones C, D, and E. For the 60-minute prediction, 9621 data points are in Zone A, 154 in Zone B, 1 in Zone C, 12 in Zone D, and 0 in Zone E. These results highlight the model’s remarkable accuracy and its capacity to reliably predict blood glucose levels.

In the 30-minute prediction (the first row of images), all data points fall within the A and B regions, indicating that the model’s predictions closely align with the device readings, exerting minimal influence on the decision-making of healthcare professionals. In the 60-minute prediction (the second row of images), only a small number of data points stray beyond the A and B regions, affirming the model’s sustained accuracy.

**Fig 6:**
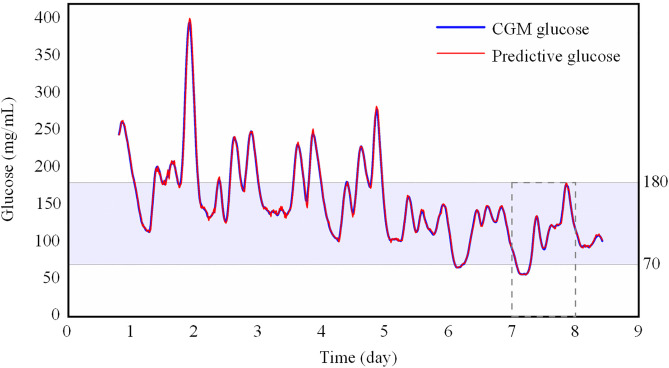
Patient’s blood glucose time profile over.

To visually demonstrate the impact of the SA-GRU model on blood glucose and adverse events prediction, we have generated a blood glucose line graph for an inpatient from the test dataset, as shown in Fig 6. This graph comprises both the predicted and CGM blood glucose values by the SA-GRU for the upcoming 30 minutes. As observed in the graph, the patient experienced hypoglycemic episodes during the nights of the 6th and 7th days of their hospitalization. This condition is well-recognized in the medical field as a perilous physiological state that can lead to severe complications. The blood glucose profile of the dotted area is shown in Fig 5.

**Fig 7:**
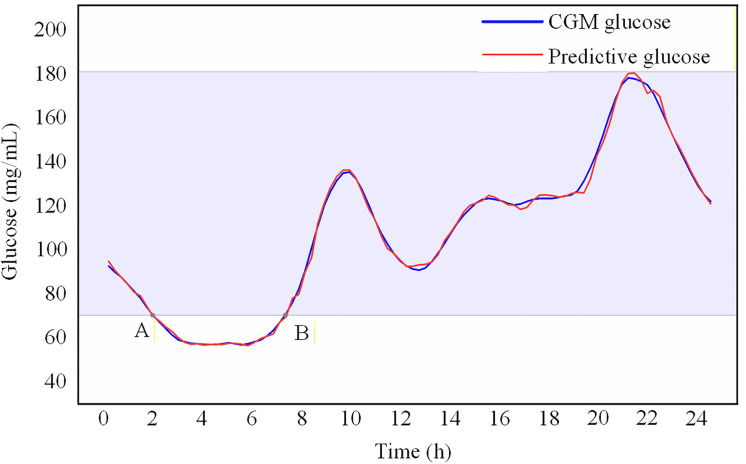
24-hour blood glucose profile. Point A is 1:57, and the blood sugar value currently is 69.97; Point B is 7:12, and the blood sugar value currently is 70.87.

Fig 7 illustrates the 24-hour blood glucose profile of the patient on day 7, and it vividly portrays the remarkable agreement between the predicted blood glucose values generated by our model and CGM values. Notably, when CGM device registered a blood glucose level of 69.97 mg/dL at 1:57, already within the hypoglycemic range, the SA-GRU model’s prediction closely aligned with a value of 69.6 mg/dL, displaying remarkable proximity to CGM data. Without factoring in delays, such as data transmission time, the model’s prediction indicated the patient’s hypoglycemia at 1:27, affording the healthcare professionals a crucial 30-minute window for timely intervention. The provision of time-sensitive information by the model plays a pivotal role in enabling prompt medical intervention to avert severe complications arising from hypoglycemia.

### 3.3 Distribution of absolute prediction errors

**Fig 8:**
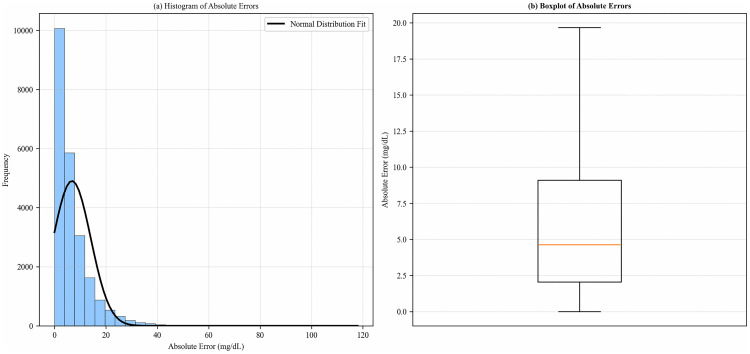
Distribution of absolute prediction errors.

To further quantify the statistical characteristics of the model’s prediction errors, we analyzed the distribution of absolute prediction errors (Fig 8). Subplot (a), the histogram, shows that errors are mainly concentrated in the low range of 0–20 mg/dL, and the fitted normal distribution curve has a certain degree of consistency with the actual distribution trend, indicating that errors are concentrated in most cases. Subplot (b), the boxplot, presents the following statistics: minimum (0.00 mg/dL), 25th percentile (Q1): 2.04 mg/dL, median: 4.63 mg/dL, 75th percentile (Q3): 9.09 mg/dL, maximum (117.98 mg/dL), standard deviation (7.28 mg/dL), and 95th percentile: 20.64 mg/dL. These characteristics indicate that the prediction errors of the SA-GRU model are generally controllable, but potential impacts of extreme errors on clinical decision-making still need attention. Future optimizations could be achieved through error calibration or clinical rule constraints.

### 3.4 Model interpretation

To enhance the interpretability of our model, we analyzed the attention weights related to both blood glucose levels and time. This analysis provides insight into how the model prioritizes certain time points and glucose values, improving its transparency and clinical relevance.

**Fig 9:**
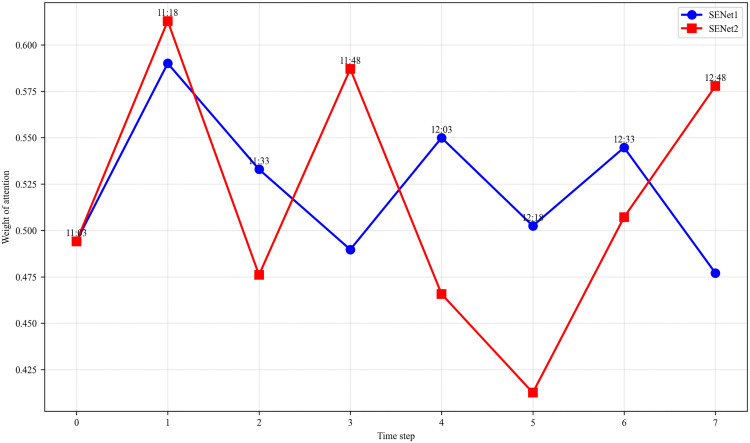
Attention weights and time relationship.

Fig 9 illustrates how the model prioritizes specific time points, offering a clear view of how temporal factors influence prediction accuracy. Notably, time points such as 11:18, 11:48, and 12:48, which correspond to higher attention weights, align with clinically significant windows—likely corresponding to postprandial periods. For instance, 11:48, which occurs approximately one hour after lunch, is a critical time for assessing postprandial blood glucose responses. This aligns closely with clinical practice, where clinicians focus on postprandial glucose trends to evaluate therapeutic efficacy and predict short-term risks, such as hyperglycemia. The model’s emphasis on these time points, consistent with clinical monitoring practices, strengthens its interpretability and clinical relevance.

**Fig 10:**
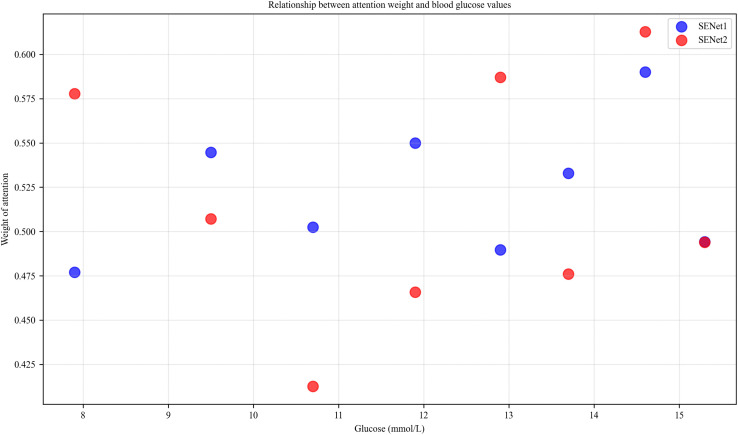
Attention weights and glucose values relationship.

Fig 10 highlights the model’s focus on extreme glucose patterns. Higher attention weights are consistently assigned to hyperglycemia (≥12 mmol/L), underscoring the model’s prioritization of critical glucose levels. In clinical practice, hyperglycemia is a key predictor of adverse events in hospitalized patients with type 2 diabetes, such as diabetic ketoacidosis and osmotic diuresis. The model’s focus on these elevated glucose values directly mirrors clinical priorities—identifying and predicting risks linked to high blood glucose levels. This consistency reinforces the notion that the model is leveraging medically significant patterns to guide its predictions, validating its clinical utility.

## 4 Discussion

This paper presents an intelligent glucose management model for hospitalized patients with type 2 diabetes, aiming to assist physicians in decision-making. The model predicts short-term blood glucose levels and adverse events based on data collected every two hours, and was trained and validated using datasets from two distinct sources. Traditionally, many studies have relied on individual blood glucose data for predictions. Typically, continuous glucose data from each patient is split into training and test sets using a predetermined ratio, such as 7:3. For instance, a patient’s 10-day glucose data might be divided, with the first 7 days used for model training and the remaining 3 days for testing.. The OhioT1DM dataset, a valuable resource, comprises dietary information from 12 patients with type 1 diabetes, insulin dosage details, and blood glucose values from 12 type 1 diabetic patients, encompassing 7 males and 5 females. This dataset was similarly divided into training and test sets at a ratio of 3:1 for each patient, facilitating model training and testing. Numerous studies have leveraged the OhioT1DM dataset to devise personalized blood glucose prediction techniques, all with the shared objective of delivering more precise glucose forecasts tailored to the unique characteristics of each patient [35–40]. Furthermore, Kim et al. [41] took an individualized approach to prediction, utilizing a RNN with data from 20 hospitalized patients diagnosed with type II diabetes. In this instance, the authors partitioned an individual patient’s blood glucose data into training and test sets at a 7:3 ratio. They then employed the patient’s historical blood glucose values to predict blood glucose levels for the subsequent 30 minutes, resulting in a RMSE of 21.5 mg/dL for the 30-minute blood glucose predictions. Mario et al. [42] adopted a LSTM-RNN for personalized blood glucose level predictions among patients with type I diabetes mellitus. They sourced data from the Artificial Intelligence Diagnosis Assistance(AIDA) simulator and the D1NAMO dataset, which includes information on dietary habits, insulin dosage, and blood glucose levels. The data from both sources were divided into training and validation sets at ratios of 7:3 and 8:2, respectively. The RMSE for 30-minute glucose predictions on both types of data was 3.45 mg/dL and 6.42 mg/dL, respectively. Li et al. [43] introduced a convolutional recurrent neural network (CRNN) model employing one-dimensional convolution and LSTM for glucose prediction in type I diabetes patients. This study drew data from two distinct sources: insulin and dietary data from 10 patients generated by the UVA/Padova simulator were used to train the model, while data from 10 patients involved in a clinical trial, of which 50% was allocated for training and the remainder for testing. The model achieved glucose predictions with RMSE of 21.07 mg/dL at 30 minutes and 33.27 mg/dL at 60 minutes. In personalized prediction methods, these models typically excel in grasping the dynamic trends within each patient’s glucose profile. Nevertheless, ensuring the model’s accuracy necessitates a substantial volume of training data for each individualized model, with training samples constituting at least 50% of the total dataset [41].

In contrast, the SA-GRU model uses a population-based training approach and does not require large amounts of individualized data. Remarkably, it can predict blood glucose levels and adverse events using just two hours of CGM data. This is especially critical in the early stages of hospitalization, where rapid treatment adjustments are necessary due to increased risks of blood glucose fluctuations and adverse events. Consequently, the SA-GRU model stands ready to offer invaluable support during these critical phases.

Our study population consists of hospitalized patients with type 2 diabetes. Due to ethical constraints and privacy issues associated with medical data, there is currently no publicly available dataset for hospitalized patients with type 2 diabetes on the internet. To validate the effectiveness and performance of the model, we conducted experiments using a dataset of non-hospitalized patients with type 2 diabetes with similar features. From a quantitative performance perspective, taking the SA-GRU model proposed in this study as an example, its RMSE for 30-minute blood glucose prediction is 4.27 mg/dL on the hospitalized Gaotang dataset (Table 4), while the RMSE on the non-hospitalized Shanghai dataset is 13.56 mg/dL (Table 6), a discrepancy directly related to the characteristic differences between hospitalized and non-hospitalized patients: in terms of treatment regimens, hospitalized patients receive real-time medication and dosage adjustments from doctors to maintain blood glucose within a fixed range, whereas non-hospitalized patients follow relatively fixed treatment plans, mostly adhering to prescribed medications. Additionally, differences in treatment protocols and dietary habits between Gaotang (Northern China) and Shanghai (Southern China)—such as hospitalized patients having fixed meal times and light diets, while non-hospitalized patients have more flexible eating patterns—further exacerbate this performance gap. To further dissect the nature of this difference, we analyzed the raw distribution of errors in the non-hospitalized dataset (Fig 8), and the results show that the median of absolute errors is 4.63 mg/dL with the 95th percentile at 20.64 mg/dL; although these values are higher than the corresponding metrics of the hospitalized dataset, they still fall within the clinically acceptable range (it is generally considered that a blood glucose prediction error < 25 mg/dL is practically valuable), indicating that while the model’s performance degrades in non-hospitalized scenarios, its basic generalization ability has been verified, and it also suggests that incorporating clinical variables such as medication adherence and dietary patterns of non-hospitalized patients is expected to further narrow the performance gap between datasets. In summary, the primary reasons for the inconsistent performance results lie in the differences in treatment and management between hospitalized and non-hospitalized patients, as well as regional variations in diet and treatment protocols, and the analysis of error distribution also points the way for subsequent model optimization.

Unlike the exclusive use of CGM data, several studies have incorporated additional variables, such as dietary and exercise information [38,42–45]. While these variables improve the authenticity and completeness of the data, they also provide a more comprehensive dataset that enhances the performance of blood glucose prediction models. However, it’s worth noting that quantifying dietary data units in real-world data collection can be a relatively intricate task. Food composition, portion sizes, and timing of intake are influenced by a multitude of variables, complicating accurate data collection. Moreover, dietary records may be susceptible to missing entries or human interference, potentially leading to data gaps that could, in turn, impact the model’s accuracy. The stability of hospitalized patients’ lifestyles, dietary intake, and exercise levels is relatively fixed. This is crucial for our research, as the model requires a stable environment and data sources to ensure its performance and accuracy. The stability of hospitalized patients makes their blood glucose data easier to control and analyze, aiding in more accurately predicting blood glucose changes and adverse events.

To mitigate the influence of extraneous factors on model accuracy, this study deliberately focused exclusively on CGM data, encompassing blood glucose values and time. Notably, the CDSS boasts a wealth of untapped data, including treatment regimens and other disease-related information. Unlike dietary data, these parameters are typically more stable and less susceptible to external influences. In forthcoming studies, it is anticipated that the inclusion of these additional data types into the model will likely result in heightened prediction accuracy.

There is some variation between the predicted and CGM blood glucose values. This error can be caused by a few factors, including the accuracy of sensor calibration, physical factors (e.g., temperature, blood fluidity), and individual differences. In addition, CGM readings can be 5–20 minutes later than the true value, and this error increases when there is a drastic change in blood glucose concentration [46]. Our model effectively predicts blood glucose levels and adverse events for the next 30 and 60 minutes, playing a crucial role in assisting medical decision-making. Despite potential delays of up to 20 minutes in CGM readings, our model still allows doctors a window of 10 and 40 minutes to take action, which is sufficient for medical interventions. With the continuous advancement of CGM-related technologies, it is anticipated that the accuracy of CGM will improve over time.

Our study aims to address the challenges of blood glucose management in hospitalized patients with diabetes, particularly focusing on real-time monitoring and adverse event prediction. By leveraging CGM data and a deep learning model, we aim to provide clinicians with a powerful tool for detecting abnormal blood glucose fluctuations and predicting potential hypoglycemic or hyperglycemic events, enabling timely interventions to reduce adverse outcomes. This objective is crucial in improving clinical care for diabetes patients and enhancing hospital management efficiency.

### 4.1 Limitations

Despite the promising predictive performance of our model on the datasets and its demonstrated clinical potential in blood glucose and adverse event prediction for hospitalized patients with type 2 diabetes, several core limitations remain in its practical clinical application. First, the model relies entirely on high-quality CGM data, which may be unavailable or inaccessible in certain healthcare settings such as primary hospitals and community health service centers; furthermore, inherent delays in CGM readings may further amplify prediction errors during rapid blood glucose fluctuations. Second, the current model only takes blood glucose values and time as input features, failing to incorporate key clinical variables such as medication use (e.g., insulin dosage adjustments), comorbidities (e.g., hypertension, hyperlipidemia), and dietary patterns. This limitation makes it difficult for the model to accurately capture dynamic changes in scenarios involving sharp blood glucose fluctuations (e.g., rapid postprandial hyperglycemia or hypoglycemia after medication administration), which is a major cause of extreme errors. Third, the study is based on retrospective observational data; while 10 repeated validations were used to reduce the impact of randomness, the model still cannot fully simulate dynamic clinical intervention processes in real-world practice (e.g., doctors adjusting treatment plans based on patients’ real-time conditions). Additionally, the study population is limited to hospitalized patients, whose disease severity and treatment standardization are relatively concentrated, potentially introducing selection bias that affects the model’s generalizability to broader populations. Fourth, addressing class imbalance remains an important aspect of the adverse event classification task, which we plan to tackle in future work. Finally, although extensive validation and evaluation were conducted using multiple datasets, the model’s universality requires further verification with real-world data from more hospitals, especially across different regions and patient groups.

### 4.2 Future directions

To address the identified limitations and promote the clinical translation of the model, we outline the following key future research directions. First, we will focus on the standardized integration and fusion of multi-source clinical features: moving beyond the single reliance on CGM data in current blood glucose prediction models, we will explore the integration of multi-source clinical variables such as medication time series, structured comorbidity information, and quantitative dietary pattern data. Through multimodal feature fusion technology, we aim to uncover potential correlations between variables and improve the model’s predictive accuracy during blood glucose fluctuations. Second, we will conduct multi-center, large-sample prospective studies: retrospective data cannot avoid selection bias or the lack of dynamic clinical intervention simulation, so future research will prioritize prospective validation involving multiple centers. The study design will cover both hospitalized and non-hospitalized populations, synchronously collecting CGM data, real-time clinical intervention records, and core prognostic indicators to objectively assess the model’s practicality and stability in real diagnostic and treatment workflows, thereby providing high-quality evidence for clinical translation. Third, we will advance the clinical integration of the model into CDSS: referring to relevant guidelines from the FDA and NMPA, three key points will be emphasized—proving clinical effectiveness through prospective studies, ensuring encrypted storage and transmission of medical data in compliance with HIPAA and the Personal Information Protection Law, and enhancing algorithm interpretability (e.g., through attention weight visualization and feature contribution analysis) to clarify prediction logic and gain trust from clinicians and regulatory approval. Fourth, we will expand the model’s applicable scenarios and optimize adaptability: to reduce reliance on high-end monitoring equipment, we will explore hybrid feature modeling combining CGM data with routine biochemical indicators, making the model suitable for resource-limited settings such as primary care. Additionally, developing lightweight model architectures (e.g., model compression, edge computing adaptation) will be a key focus to meet the real-time decision-making requirements of CDSS and promote the model’s implementation in a wider range of clinical scenarios. Finally, we envision deep integration of the model into real-world hospital workflows through connection with clinical information systems (CIS) or electronic medical record (EMR) systems: predictive results will be displayed in real time on clinicians workstations, serving as a decision-support tool—for example, automatically alerting healthcare professionals to take necessary interventions when high risks of hypoglycemia or hyperglycemia are predicted. This real-time feedback will assist clinicians in formulating personalized treatment plans, avoiding significant blood glucose fluctuations or acute complications, thereby improving patient care and alleviating the overall burden on healthcare systems by preventing costly complications and readmissions.

## 5 Conclusion

We propose a deep learning model for managing blood glucose levels in hospitalized patients with type 2 diabetes. This model predicts blood glucose values and adverse events for the next 30 and 60 minutes based on two hours of CGM data. The model demonstrated low error in glucose prediction and high accuracy in adverse events prediction. Once integrated into the CDSS, healthcare professionals will have real-time access to patients’ future glucose levels and adverse event predictions, enabling timely intervention. This model serves as a practical decision support tool for healthcare professionals and offers an actionable approach to optimizing inpatient blood glucose management.
